# Recent advances in managing primary hypertension

**DOI:** 10.12703/b/9-4

**Published:** 2020-11-04

**Authors:** Ioannis Leontsinis, Manos Mantzouranis, Panagiotis Tsioufis, Ioannis Andrikou, Costas Tsioufis

**Affiliations:** 1 First Cardiology Clinic, Medical School, National and Kapodistrian University of Athens, Hippokration Hospital, 108 Vas. Sofias Ave, 11527, Athens, Greece

**Keywords:** hypertension, novel antihypertensives, neuromodulation, resistant hypertension, hypertension management, difficult to treat hypertension

## Abstract

Hypertension remains a leading risk factor for cardiovascular mortality and morbidity globally despite the availability of effective and well-tolerated antihypertensive medications. Accumulating evidence suggests a more aggressive blood pressure regulation aimed at lower targets, particularly for selected patient groups. Our concepts of the optimal method for blood pressure measurement have radically changed, maintaining appropriate standard office measurements for initial assessment but relying on out-of-office measurement to better guide our decisions. Thorough risk stratification provides guidance in decision making; however, an individualized approach is highly recommended to prevent overtreatment. Undertreatment, on the other hand, remains a major concern and is mainly attributed to poor adherence and resistant or difficult-to-control forms of the disease. This review aims to present modern perspectives, novel treatment options, including innovative technological applications and developing interventional and pharmaceutical therapies, and the major concerns emerging from several years of research and epidemiological observations related to hypertension management.

## Introduction

**Figure 1.**
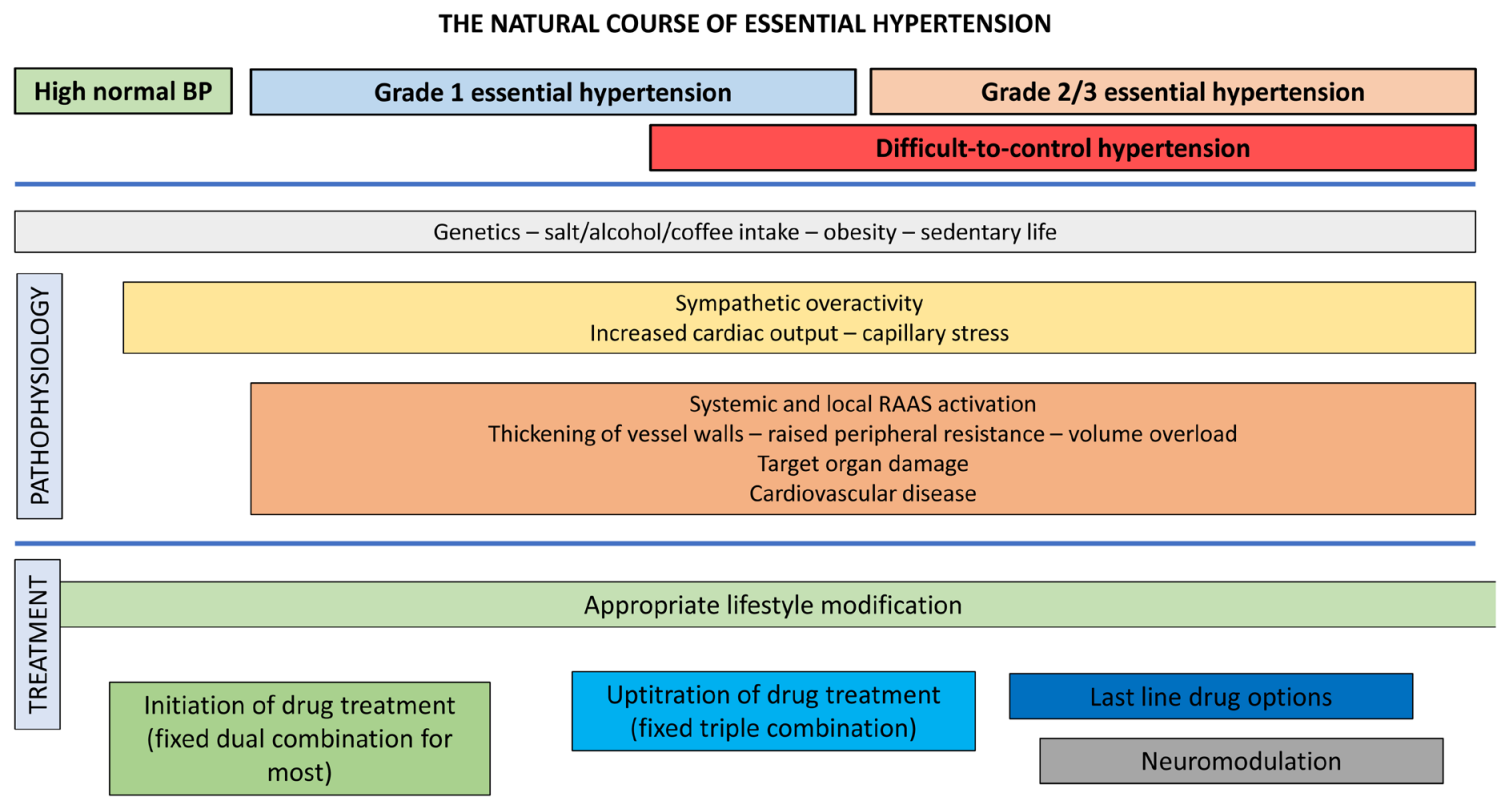
The natural course of essential hypertension. BP, blood pressure; RAAS, renin angiotensin aldosterone system.

Hypertension (HTN) constitutes a leading risk factor for cardiovascular morbidity and mortality worldwide. It refers to the condition of sustained elevated blood pressure (BP) levels, and it affects all ages, both genders, and every ethnicity^1^. Usually there is no single identifiable causative condition. This is the commonest scenario, referred to as primary HTN (Figure 1). Its global prevalence based on office BP measurements reached 1.13 billion people in 2015. With an estimated overall prevalence of 30–45% among the adult population, it is addressed as a major public health burden^1^.

Forounzanfar *et al*. showed that elevated BP (defined as systolic BP of at least 110–115 mmHg) was the leading global contributor to premature death in 2015, accounting for almost 10 million deaths and over 200 million disability-adjusted life years^2^. Importantly, despite the increasing public awareness and several significant advances in diagnosis and treatment, the disability-adjusted life years attributable to HTN have increased by 40% over the last four decades. Systolic BP ≥140 mmHg accounts for the majority of the mortality and morbidity burden (~70%). In more detail, the largest number of systolic BP-related deaths per year were attributed to ischemic heart disease (4.9 million), hemorrhagic stroke (2.0 million), and ischemic stroke (1.5 million)^2^. This review aims to present some of the modern perspectives, treatment options, and concerns emerging from several years of research and epidemiological observations related to HTN management. Secondary HTN corresponds to a minority of hypertensive subjects and is attributed to specific and potentially curable causes; however, this topic will not be discussed here.

## Hypertension definition and treating targets: shifting to lower levels

Currently, the definition of HTN differs across the Atlantic^3^. The 2018 European Society of Cardiology/European Society of Hypertension (ESC/ESH) guidelines continue to support the traditional threshold of office BP levels above 140/90 mmHg for the definition of HTN^1^, whereas the 2017 American College of Cardiology/American Heart Association (ACC/AHA) ones set the threshold of 130/80 mmHg^4^. Suboptimally controlled HTN has been consistently associated with worse cardiovascular outcomes^1,4^. Nevertheless, overtreating HTN can cause serious adverse events^5,6^. Epidemiological data provide incremental evidence regarding optimal BP control, creating an evolving substrate for continuous consideration. Thus, taking into consideration the high BP variability particularly associated with office BP measurements, the range of BP levels recommended by the European guidelines may better approach the ideal treatment targets.

There is accumulating evidence regarding the benefit of aggressive BP lowering in selected groups. The landmark SPRINT study^7^ targeting lower BP levels in non-diabetic hypertensives showed reductions in all-cause mortality, cardiovascular events, and indices of cerebrovascular disease progression^8–11^. A post-hoc analysis of the ACCORD-BP results showed the same beneficial effect when glycemia was addressed conservatively^12,13^. Registries with longer follow up duration show almost unanimously that even after the discontinuation of antihypertensive treatment, most of the enrolled hypertensive subjects seem to sustain benefit in terms of cardiovascular prognosis, a phenomenon addressed as the “legacy effect” of the antihypertensive treatment in literature. Most of these registries controlled the efficacy of the intensity (different treatment targets, i.e. HDFP, UKPDS) or the quality (different treatment options, i.e. ASCOT, ALLHAT) of the offered treatment. It seems that more intensive and/or efficient treatment is related to fewer cardiovascular events over time^14^.

Many investigators argue that the SPRINT results cannot guide decision making for the whole population^15^. Of particular concern are frail subjects, who are prone to side effects from overtreatment. A large-scale meta-analysis of several randomized clinical trials (RCTs) suggested clearly a higher BP target for older patients^16^. Considering the higher risk of dehydration, orthostatic hypotension, acute kidney injury (AKI)^17^, or syncope, among subjects treated intensively in SPRINT, the skepticism grows^18^. Nevertheless, secondary analyses bring further evidence favoring the intense approach. Byrne *et al*. supported the notion that risks and benefits attributed to the intensive treatment were not affected by age^19^. Additionally, seniors at high risk of developing treatment-related side effects (i.e. dehydration, orthostatic hypotension) seemed to benefit from intensive BP treatment^20^. Orthostatic hypotension was not found to be related to higher risk for cardiovascular events. Although associated with hypotension-related hospitalizations and bradycardia, this risk was similar in the different arms of the study^21^. Finally, hemodynamic effects rather than tubular dysfunction are the proposed etiology for AKI cases^22^. A similar hypothesis has been derived from the ACCORD-BP results^23^.

Apart from frailty, there are concerns regarding subjects with low cardiovascular risk. To date, studies enrolling low-cardiovascular-risk hypertensives failed to demonstrate significant benefit in outcomes derived from BP lowering per se^24,25^. A post-hoc analysis of the SPRINT results showed that the beneficial effects of intense BP lowering were sustained in variable cardiovascular risk^26^. However, SPRINT did not enroll low-risk subjects^27^.

## Blood pressure measurement and monitoring

BP constitutes one of the most commonly evaluated biological parameters influencing decision-making in various clinical settings. Direct measurement via intra-arterial catheters remains the most accurate way; however, its invasive nature keeps it limited to critically ill patients or intraprocedural monitoring. Indirect measurement with automatic sphygmomanometers is the preferred method for the majority of situations. The accuracy and reliability of such validated devices is increasing, and their usage is widespread for both stationary and ambulatory measurements. Arrhythmias degrade their accuracy^28^; however, innovative technologies are targeting higher standards. On the contrary, the manual technique, because of its high intra- and inter-observer variability, is constantly losing ground.

Ambulatory BP monitoring (ABPM) plays a central role, unveiling patients with masked or white-coat HTN and giving information regarding sleep-dipping status or enhancing the discrimination of true resistant hypertensive patients. Together with home BP monitoring (HBPM), they constitute a better predictor for HTN-mediated organ damage compared to office stationary BP checks. However, the available targets for treatment rely upon office BP readings, as there are missing data for the out-of-office ones^1^.

The circumstances under which office BP readings should be obtained remain controversial. The absence of any health professional during the procedure, known as the unattended technique, is believed to attenuate the white-coat phenomenon so that values are lower than the conventional attended method, an observation supported by several studies^29^. Currently, the literature suggests that both techniques are well correlated with HTN-mediated organ damage^30^. Additionally, the beneficial effect of intensive treatment in SPRINT does not seem to be related to the applied technique^31^. In view of the need for a homogenous approach, the methodology of the measuring technique is crucial. Further studies are warranted to establish which method is more cost effective.

Evidence is growing regarding the anticipated benefits from remote BP monitoring techniques^28^. Big data collection, by means of wearable biosensors, may enable artificial intelligence applications to search for new insights on HTN pathogenesis and biomarker development or to evaluate the efficacy of the applied treatment^32–34^. In this direction, the cuff-less continuous BP monitoring concept is gaining traction. Photoplethysmography (PPG) has been used to monitor other biological parameters; however, its usage in BP measurement remains under investigation^35^. BP self-monitoring and telemonitoring demonstrate efficiency to increase adherence to medication and improve outcomes. In view of cost-effectiveness, self-monitoring is currently suggested as a less-expensive and equally efficient strategy^36^.

## Blood pressure variability: a potential adjunctive treatment target

Recent data imply that homogenous 24-hour BP control could further protect from target-organ damage progression and reduce cardiovascular morbidity. Stronger evidence coming from RCTs is warranted to support the incorporation of BP variability treatment targets in clinical practice^37,38^.

## Neurovascular dimension in hypertension: neuromodulation

The role of the sympathetic nervous system in cardiovascular homeostasis and dysfunction is well recognized yet not fully understood. Neuromodulation refers to pharmaceutical or interventional techniques which aim to restore or downregulate the sympathetic outflow transmission in different levels.

Previously, the pharmaceutical industry targeted several loci to attenuate the sympathetic overdrive in HTN. These efforts concern the usage of adrenergic blockers or centrally acting drugs, accompanied by different levels of efficacy and significant side effects, as they lack specificity on peripheral tissues. Recent laboratory and pre-clinical results highlight another approach. ADRQβ-004, a vaccine for the alpha-1D adrenergic receptor, demonstrated significant antihypertensive action in animal models^39^. Similarly, allopregnanolone, a neurosteroid agent, was found to reduce BP via selective modulation of gamma-aminobutyric acid-a receptors^40^.

The interplay between autonomic dysfunction and inflammation is crucial^41^. Endothelial dysfunction has been strongly correlated with vascular aging, arterial stiffness, and high BP. Angiotensin II constitutes a key molecule in this process^42^. Recent findings connect the baroreflex imbalance with inflammation beyond the blood–brain barrier (BBB). It has also been suggested that BBB integrity is impaired in hypertensive subjects, and this condition promotes autonomic dysfunction^43^. Astroglial dysfunction, renin angiotensin system (RAS) hyperactivation, oxidative stress, and inflammatory cytokines include some of the proposed mediators of neuroinflammation, which in turn alter the function of several central nervous cardioregulatory centers, causing HTN^44^. Recently invented molecules exert modulatory effects on neuroinflammation. Firibastat was found to lower BP levels by reducing brain angiotensin III and vasopressin release^45^. Minocycline is under investigation for resistant HTN owing to its properties to inhibit microglia activation^44^.

Device-based interventions comprise the second main pillar of neuromodulation and constitute a highly expanding field in HTN treatment. Many different attempts have been made to explore potential techniques to interrupt or regulate the sympathetic influence on systemic vasculature and target organs^46^.

Catheter-mediated renal denervation (RDN) constitutes the most extensively investigated interventional technique. Carotid baroreceptor stimulation and arteriovenous fistula formation have also been tested in HTN treatment; however, these options will not be discussed in the present review. Early RDN studies using radiofrequency ablation demonstrated significant BP reductions among subjects with resistant HTN. Despite the discouraging SIMPLICITY HTN-3 trial, recent data from second-generation sham-controlled studies bring RDN back into the spotlight, particularly by confirming the safety of the approach and unequivocally demonstrating beneficial short-term effects on BP control in subjects with mild, moderate, or resistant HTN^47–50^. A smaller cohort demonstrated similar efficacy in end-stage renal disease patients^51^. The aforementioned positive results can be attributed to several factors^52^. Increasing operator experience, newly acquired knowledge regarding renal artery anatomy^53^, improved study designs, and the incorporation of novel catheters enabling ablation at multiple sites are part of this list. Interestingly, findings from various studies have expanded the scope of RDN beyond HTN treatment. ERADICATE-AF researchers, with the limitation of not using the sham control method, supported the finding that RDN, when combined with atrial fibrillation ablation, improved outcomes^54^.

Specialists are currently reservedly optimistic in anticipation of the long-term follow up from major RDN RCTs. The SPYRAL HTN-OFF MED pilot trial was an international, randomized, single blind, sham control trial which tested RDN in patients with uncontrolled HTN. Despite the limited-duration follow up of three months, the study managed to provide biological proof of principle for the BP-lowering efficacy of the procedure^55^. The recently published SPYRAL-OFF MED Pivotal trial (SPYRAL Pivotal) demonstrated a statistically significant and clinically relevant office and ambulatory BP reduction in the absence of antihypertensive medications compared with a sham control group^56^. The SPYRAL HTN-ON MED trial had a similar design but tried to test RDN as a therapy adjunctive to medical treatment in humans with moderate HTN. Results from the first 80 participants showed that RDN of the main renal arteries and branches significantly reduced BP levels as compared with sham groups with no safety concerns^49^.

Although promising, challenges regarding RDN technique are still present. Identification of specific patient characteristics determining responsiveness to the procedure is one of them. These include aspects such as the renal artery’s anatomical properties, patients’ comorbidities which greatly affect sympathetic tone (i.e. diabetes and obesity), and sensitivity to salt intake. In addition, there are still considerations to be made regarding procedural improvements needed to enhance the acquired results. In this direction, there is evidence supporting the view that the outcome improves as the treated area increases in circumference and depth^57^. However, the presence of a sensitive biomarker which would allow the operator to assess the treatment result interprocedurally is still missing^57^. Nevertheless, in our opinion, we are getting closer than any time before in addressing the technique as a lifetime intervention for HTN. Current guidelines to date preserve it for the environment of clinical studies, a perception which may change in the near future.

## Novel antihypertensive drugs under development

### Renin angiotensin aldosterone system

Non-steroidal mineralocorticoid receptor (MR) antagonists exhibit high selectivity and increased affinity for the MR without interfering with progesterone and androgen receptors. They show higher potency and increased half-life than eplerenone without the anti-androgenic effects of spironolactone^58^. In the ARTS-DN trial, finerenone demonstrated reduced hyperkalemia risk but limited utility as an antihypertensive^59,60^. Esaxerenone recently showed promising results in a phase III RCT (ESAX-HTN) compared to eplerenone regarding safety and hypertensive efficacy^61^.

Aldosterone synthase inhibitors inhibit CYP11B2 (aldosterone synthase), reducing the aldosterone and renin circulating levels. First-in-class drug LCI699 was abandoned, as it was shown to inhibit concomitantly CYP11B1, resulting in a compensatory increase of adrenocorticotropic hormone. Current efforts are focusing on the development of more selective agents^60,62^.

An alternative RAAS pathway leading to vasodilatation and natriuresis was recently discovered. It is mediated by the conversion of angiotensin II to angiotensin (1,7) via angiotensin-converting enzyme (ACE) 2. ACE2 activators, angiotensin (1,7) agonists, and angiotensin receptor 2 (AT2) agonists comprise potential targets of novel antihypertensive drugs with negative results so far^60,63^. Firibastat, a centrally acting aminopeptidase A inhibitor, lowered BP levels in the NEW-HOPE study^60,64^.

### Endothelin pathway

Endothelin-1 (ET-1) is secreted by endothelial cells after the cleavage of inactive big ET-1 by endothelin-converting enzyme (ECE)^65^. It exerts its vasoactive actions by binding to endothelin type-A receptors (ETAs) and endothelin type-B receptors (ETBs). ETAs are primarily found in vessels, and their activation induces potent vasoconstriction. On the contrary, binding of ET-1 on ETB is associated with vasodilatation^66^. Based on these data, research in the specific field investigated ET-1 receptors and ECE as potential therapeutic targets^67^.

ET-1 receptor antagonists (ERAs) were initially developed for the treatment of pulmonary arterial HTN (PAH). From an early stage, there were hopes for their expansion in HTN. A recent meta-analysis of 18 studies including 4,898 hypertensive patients confirmed a significant reduction of office and 24 hour ABP monitoring related to ERAs at the cost of increased severe adverse effects^68^. Darusentan was investigated for use in resistant HTN with unfortunately discouraging results, which were mainly attributed to methods of BP measurement^65,66^. However, aprocitentan, a new dual ETA/ETB antagonist, showed encouraging results in a phase II study compared with lisinopril and placebo^69^. An ongoing phase III study (PRECISION) will evaluate its role in resistant HTN (NCT03541174).

### Dual-acting RAS–neprilysin inhibitors

Neprilysin (NEP) is a metalloprotease responsible for the degradation of atrial natriuretic peptide and brain natriuretic peptide^60^. Efforts to develop an antihypertensive NEP inhibitor did not flourish because of concomitant degenerative effects on vasoconstrictive peptides such as angiotensin II and endothelin^65^. Thus, research proceeded on dual pathways, combining NEP and RAS inhibition together. Omapatrilat, a dual NEP–ACE inhibitor, exhibited significant BP-lowering potency, but safety concerns (angioedema) suspended its development. The reduced risk of angiotensin receptor blockers (ARBs) to cause angioedema led researchers to develop LCZ696. A dual AT2–NEP inhibitor (ARNI), a combination of sacubitril and valsartan, was recently established as a treatment for heart failure with reduced ejection fraction (HFrEF). Tested in several studies, its efficacy on BP reduction is well documented^60,70,71^. Moreover, a meta-analysis of 11 RCTs concluded that ARNIs are more effective against HTN compared with ARBs alone while showing similar safety^72^.

Despite the aforementioned impressive data, sacubitril has been linked with increased generation of beta-amyloid *in vitro*, increased levels of which are observed in the cerebrospinal fluid of patients with Alzheimer’s disease^73^. Recent studies assessing the potential neurotoxic side effects of LCZ696 in therapeutic doses were negative^74^; however, the incorporation of the drug in the treatment of primary HTN would require higher doses and longer exposure^75,76^. If concerns for potential cognitive impairment persist, it seems that the future use of LCZ696 as an anti-hypertensive will be reserved for older, resistant HTN^77^ and patients with HFrEF.

### Sodium-glucose cotransporter-2 inhibitors

These are novel oral hypoglycemic drugs that inhibit the renal reabsorption of glucose in the proximal tubule^78^. Major studies of all three approved sodium-glucose cotransporter-2 inhibitors (SGLT2is)—empagliflozin, canagliflozin, and dapagliflozin—were landmarks in the new era of cardioprotective and nephroprotective anti-diabetic drugs owing to these agents’ impressive class-effect outcomes regarding cardiovascular and all-cause mortality, hospitalizations for heart failure, and progression of diabetic nephropathy^79^.

Besides offering glycemic control and a favorable cardiorenal impact, SGLT2is demonstrated a modest but significant decrease of BP levels in all relevant studies^80^. However, as confirmed by recent meta-analyses, the level of BP reduction is slight^81–83^. Regarding the potential side effects, major concerns have been raised because of the higher incidence of urinary tract infections, diabetic ketoacidosis, fractures, and limb amputations noticed in some of the main trials of SGLT-2is^84,85^. Two major ongoing studies are currently addressing the antihypertensive effect of dapagliflozin (NCT01195662) and canagliflozin (NCT01939496)^78^.

### Other agents

***Angiotensinogen small interfering RNAs*.** Small interfering RNAs (siRNAs) are molecules that silence the translation of selected target mRNAs. Inclisiran inhibits the translation of proprotein convertase subtilisin/kexin type 9 mRNA in hepatocytes, offering a long-term sustained reduction of LDL^86^. Likewise, an angiotensinogen siRNA developed in an effort to inhibit the renin angiotensin aldosterone system (RAAS) at its roots has shown promising results in an animal study^87^.

***Phosphodiesterase type 5 inhibitors*.** Phosphodiesterase inhibitors negate the degenerative effect of cGMP-specific phosphodiesterase type 5 (PDE5) on cyclic GMP in the smooth muscle cells of the blood vessel wall, thus exerting a potent vasodilatory impact. PDE5 inhibitors have been established in the treatment of erectile dysfunction and PAH and remain under investigation for their potential utility in HTN based on a solid pathophysiological substrate^88^.

***Nitric oxide donors and nitrates*.** Organic nitrates, such as nitroglycerin, offer potent vasodilatory properties, but their brief half-life and frequent side effects limit their long-term clinical utility. On the other hand, dietary sources of inorganic nitrates, such as beetroot juice, are under investigation as an affordable, add-on choice in the treatment of HTN^89^.

***Microbiota-targeted therapy, vaccines, and nutraceuticals*.** Recent experimental data have associated alterations in gut microbiome caused by high salt consumption with the activation of Th17 lymphocytes, which in turn are believed to promote autoimmunity and HTN. Preliminary data have identified Lactobacillus as a “natural inhibitor” of the high-salt environment activation of TH17 cells^90^.

AngQb was a promising vaccine against angiotensin II that has already completed a phase IIa trial in patients with primary HTN and demonstrated significant reduction of ABP without raising major safety issues. Other vaccines targeting angiotensin II and angiotensin II receptors are currently being investigated, such as the angiotensin II DNA vaccine (AGMG0201)^63,65,91^.

Many dietary ingredients have been shown to lower BP levels via different pathways^92–97^. To date, there are no available data from RCTs to support the beneficial effects of such nutraceuticals in HTN treatment.

Other interesting molecules under preliminary investigation are natriuretic peptide receptor agonists, vasoactive intestinal peptide analogues, vasopressin antagonists, intestinal Na^+^/H^+^ exchanger 3 inhibitor, dopamine β-hydroxylase inhibitors^60,63^, and carotid body purinergic P2X3 chemo-receptor blockers^98^.

### Optimal drug delivery systems

Apart from the development of novel antihypertensive drugs aiming to provide solutions in difficult-to-control HTN and non-adherence settings, researchers have also focused on the improvement of drug delivery systems. Biodegradable polyester drug delivery systems play a pivotal role in this effort. They enhance bioavailability and pharmacokinetics while offering the possibility for administration of the drug to the target tissue, preventing premature degradation^99^.

## Clinical decision-making: managing hypertension

Cardiovascular disease constitutes a well-recognized socio-economic burden in the modern era. Morbidity, mortality, and disability rates attributed to major cardiovascular events remain significantly high despite substantial progress in the field of prevention^2^. Health systems around the globe have invested a significant amount of resources in risk stratification techniques^1,4^. These focus on both primary and secondary prevention. The first refers to asymptomatic individuals and aims to detect timely subclinical forms of the disease or individuals at increased risk of developing a cardiovascular disorder. Preventing HTN is increasingly challenging. Obesity, sedentary lifestyle, poor diet, high salt intake, smoking, and related morbid conditions such as diabetes and obstructive sleep apnea in combination with an aging population make this task substantially demanding^2,100,101^. The second applies to patients with known cardiovascular disease and focuses on risk factor modification and other structured interventions imposed in order to increase life expectancy and quality of life.

### First-line treatment strategy

According to the latest guidelines, the fundamental therapeutic intervention for HTN remains drastic lifestyle modification focusing on regular physical activity, incorporation of a healthier diet, weight reduction, restriction of salt, caffeine, and alcohol consumption, and the cessation of smoking. These measures seem to delay the development of HTN and most interestingly could even replace drug treatment in patients with grade 1 HTN. Furthermore, they reduce cardiovascular risk and enhance the effectiveness of drug treatment regimens^1,102^.

### Single pill combination treatment: the new approach

The latest ESC/ESH guidelines recommend the initiation of a single pill combination (SPC) of two drugs of the five major classes, followed by an uptitration to a three-drug SPC if needed. Exceptions to consider initial monotherapy are patients with high–normal BP and high cardiovascular risk, patients with grade 1 HTN and low/moderate risk, and frail elderly patients (particularly if systolic BP is <150 mmHg)^1^. The 2017 ACC/AHA guidelines suggest initial treatment with a single drug in patients with stage 1 HTN but a two-drug combination in those with stage 2 and an average BP more than 20/10 mmHg above their BP target^103^. Drug combinations are more effective and better tolerated compared to maximal dose monotherapies because they target different mechanisms^104–106^. The SPC regimen demonstrated increased adherence to treatment and higher rate of BP control and thus is expected to improve cardiovascular outcomes^107–109^.

### Resistant and difficult-to-control HTN

Despite the progress in HTN treatment, the wide availability of different drug classes with potent combinations, and even the increasing use of SPCs, difficult-to-control HTN remains a critical issue in modern clinical practice considering the fact that these patients face an increased risk of HTN-mediated organ damage, chronic kidney disease (CKD), and cardiovascular events. Definition of resistant HTN requires the use of optimal or best-tolerated doses of at least three drugs, which should include a diuretic. Failure to achieve BP target should be confirmed by ABPM or HBPM and adherence to treatment should be taken into account in order to exclude the usual pseudoresistance scenarios. Additional main causes of difficult-to-control HTN include obesity, high sodium and alcohol intake, prescribed and non-prescribed drugs that induce HTN, forms of undiagnosed secondary HTN, and cases of advanced HTN-mediated organ damage such as CKD and large-artery stiffening. After careful assessment, the true resistant HTN prevalence is estimated at <10% of treated patients^1,110^.

For the treatment of difficult-to-control HTN, the latest guidelines recommend a sequential approach (Figure 1). Failure to achieve BP targets with uptitration or replacement of the selected diuretic should be followed by spironolactone (or amiloride) addition according to the results of the PATHWAY 2 study^111^, where they demonstrated superiority versus placebo, bisoprolol, and doxazosin in the treatment of drug-resistant HTN. Eplerenone constitutes an appropriate alternative. Bisoprolol and doxazosin are suggested as the final add-on drugs^1^. Direct vasodilators (hydralazine, minoxidil) can cause fluid retention and reflex tachycardia. If used, they should be combined with a loop diuretic and beta blocker. Centrally acting drugs appear to be a last resort owing to a less favorable pharmacological profile with significant central nervous system adverse effects, rebound HTN, and inferior effectiveness compared to spironolactone according to the ReHOT trial^103,112^.

## Conclusions

HTN is a constantly shifting clinical field. Despite the significant progress, it remains a leading risk factor for mortality and morbidity globally. In our opinion, current knowledge encourages clinicians to address treatment strategies in an individualized manner. Thorough risk stratification should always precede decision making. Lower treatment targets may have to be applied in high-risk individuals, taking into consideration potential adverse events of overtreatment. Innovative technological applications, improved interventional techniques, and novel drugs are expected to enable researchers and clinicians to address current difficulties such as poor adherence to treatment or resistant forms of the condition in a more efficient way.

## Abbreviations

ABP, ambulatory blood pressure; ACC, American College of Cardiology; ACE, angiotensin-converting enzyme; AHA, American Heart Association; AKI, acute kidney injury; ARB, angiotensin receptor blocker; ARNI, dual-acting angiotensin receptor–neprilysin inhibitor; AT2, angiotensin receptor 2; BBB, blood-brain barrier; BP, blood pressure; CKD, chronic kidney disease; ECE, endothelin-converting enzyme; ERA, endothelin-1 receptor antagonist; ESC, European Society of Cardiology; ESH, European Society of Hypertension; ET-1, endothelin-1; ETA, endothelin type A receptor; ETB, endothelin type B receptor; HBPM, home BP monitoring; HFrEF, heart failure with reduced ejection fraction; HTN, hypertension; MR, mineralocorticoid receptor; NEP, neprilysin; PDE5, phosphodiesterase type 5; RAAS, renin angiotensin aldosterone system; RAS, renin angiotensin system; RCT, randomized clinical trial; RDN, renal denervation; SGLT2i, sodium-glucose cotransporter-2 inhibitor; siRNA, small interfering RNA; SPC, single pill combination.
